# 

**Published:** 1881-05

**Authors:** 


### CONTENTS FOR MAY, 1881.

ORIGINAL COMMUNICATIONS.

ART. I.—Ether. By LAURENCE TURNBULL, M. D. .... 225

ART. II.—Gold Versus Rubber for Artificial Dentures. By DR. I. N. CARR, M. D. ... 233

ART. III.—Sacredness of the Medical Profession. By W. R. MONROE, M. D. .... 238

ART. IV.—Quinia in Antiseptic Treatment. By HUGO ERICHSON .... 239

EDITORIAL DEPARTMENT.

Publication of Medical Society Proceedings .... 241
Medical Gymnastics for the Phthisical .... 242
Our Popular Science Department. Quinine .243–244
Diploma Mills Maternite Hospitals .... 245
Correspondence .... 246
Occupations Suitable for Women. Abattoirs.246–247
Biology and Johns Hopkins University .... 247
Dr. J. Marion Sims. Dr. Eugene F. Cordell .... 248
Medical Education. The Kidd-Quain Embroglio 249
The American Medical Association .... 251
Meeting of the Medical Association of Georgia .. 253
Alabama Dental Association .... 253
Our Removal to New York .... 296

BIBLIOGRAPHICAL DEPARTMENT.

Lectures of Diseases of the Nervous System .... 254
Introduction to Pathology and Morbid Anatomy 255
The Diseases of Children .... 255
The Management of Children in Sickness and in Health .... 256
How a Person Threatened or Afflicted with Bright's Kidney Disease Ought to Live .... 256
Post-Mortem Examinations .... 256
American Health Primers .... 256
Domestic Hygiene. The Sanitary News .... 258
The Texas Medical and Surgical Record .... 258
The Monthly Review of Medicine and Pharmacy 258
The Relation of the Marine Hospital Service of United States to Commerce, the Public and Medical Profession .... 258
Report of the Committee of the Medical Society of the State of California .... 259
THERAPEUTIC PEARLS AT RANDOM STRUNG....259–261
EXCERPTA .... 261–274

POPULAR SCIENCE DEPARTMENT.

Are all the Races of Men Descended from a single Pair? .... 274
Commerce. Sleeplessness from Thought....279–280
Frequent Changes of the Underclothing .... 282
Relations of Sanitation to Christianity .... 283
Trichinæ in Man. Destruction of Forests ..284–286
Some Points on Cleansing the Ear .... 286
Sanitary Insurance. Rules for House Drainage. 288
Adulterated Coffee .... 289
Separating Gold, Silver and Copper Alloy by Electrolysis .... 290
The Bad Effects of Train-Catching .... 291
Precocity a Sign of Inferiority .... 291
Capacity for Study in Children .... 292
The Moquis. Don't Bathe After Meals.....292–293
The Weight of the Brain and its Functional Activity .... 293
The Physiology of the Visual Purple .... 294
HYMENIAL. NECROLOGICAL .... 596
Mortuary Report .... 298

